# Serum CYFRA 21-1 as a Prognostic Marker in Non-Small-Cell Lung Cancer Patients Treated with Immune Checkpoint Inhibitors

**DOI:** 10.3390/cancers16213712

**Published:** 2024-11-04

**Authors:** Keiki Miyadera, Sho Kakuto, Mayu Sugai, Ryosuke Tsugitomi, Yoshiaki Amino, Ken Uchibori, Noriko Yanagitani, Hisatoshi Sugiura, Masahiro Seike, Makoto Nishio, Ryo Ariyasu

**Affiliations:** 1 Department of Thoracic Medical Oncology, Cancer Institute Hospital of Japanese Foundation for Cancer Research, Tokyo 135-8550, Japan; s11-099mk@nms.ac.jp (K.M.); sho.kakuto@jfcr.or.jp (S.K.); mayu.sugai@jfcr.or.jp (M.S.); ryosuke.tsugitomi@jfcr.or.jp (R.T.); yoshiaki.amino@jfcr.or.jp (Y.A.); ken.uchibori@jfcr.or.jp (K.U.); noriko.yanagitani@jfcr.or.jp (N.Y.); mnishio@jfcr.or.jp (M.N.); 2 Department of Pulmonary Medicine and Oncology, Graduate School of Medicine, Nippon Medical School, Tokyo 113-8602, Japan; mseike@nms.ac.jp; 3 Department of Respiratory Medicine, Tohoku University Graduate School of Medicine, Sendai 980-8574, Japan; hisatoshi.sugiura.a4@tohoku.ac.jp

**Keywords:** CYFRA 21-1, serum tumor marker, non-small-cell lung cancer, immune checkpoint inhibitor

## Abstract

**Background:** A prognostic marker in patients with non-small-cell lung cancer (NSCLC) treated with anti-PD-1/PD-L1 antibodies must be established. This study explored serum cytokeratin fraction 21–1 (CYFRA 21-1), which represents a squamous cell histology, as a prognostic factor in anti-PD-1/PD-L1 antibody treatment, stratifying by histology and treatment regimen. **Methods:** This study retrospectively evaluated patients with advanced NSCLC without driver mutations receiving anti-PD-1/PD-L1 antibodies between November 2015 and March 2023. Cutoff values for CYFRA 21-1 and carcinoembryonic antigen (CEA) were 3.5 and 5.0 ng/mL, respectively. The Kaplan–Meier method and a log-rank test were conducted. The Cox proportional hazards model was utilized for univariate and multivariate analyses. **Results:** This study included 258 patients. The squamous NSCLC group demonstrated a shorter overall survival (OS) than the non-squamous NSCLC group (median, 17.8 vs. 23.7 months, *p* = 0.141). Patients with high serum CYFRA 21-1 and CEA levels exhibited a significantly shorter OS than those with normal levels (median, 11.7 vs. 32.7 months, *p* < 0.005; 15.8 vs. 29.7 months, *p* < 0.005). The multivariate analysis identified a performance status (PS) of ≥2, a PD-L1 expression of ≥50%, and a serum CYFRA 21-1 of >3.5 ng/mL as independent prognostic factors. Patients with high serum CYFRA 21-1 levels exhibited a significantly shorter OS even focusing on non-squamous NSCLC, anti-PD-1/PD-L1 antibody and chemotherapy combination therapy, or anti-CTLA-4 antibody combination therapy. **Conclusion:** Serum CYFRA 21-1 is a poor prognostic marker for patients with NSCLC receiving anti-PD-1/PD-L1 antibody treatment even when stratifying by histology or treatment regimen.

## Simple Summary

A prognostic marker in patients with non-small-cell lung cancer (NSCLC) treated with anti-PD-1/PD-L1 antibodies must be established. In this study, a high serum cytokeratin fraction 21–1 (CYFRA 21-1) level was found to be a poor prognostic marker in patients with NSCLC receiving anti-PD-1/L1 antibodies even stratifying by histology or treatment regimen. Thus, serum CYFRA 21-1 may be useful for precision medicine with anti-PD-1/PD-L1 antibody treatment.

## 1. Introduction

Recently, the survival rates of patients with lung cancer has tended to increase [1,2,3]. Immune checkpoint inhibitors (ICIs) are an advanced therapy because they enable achieving long-term responses. However, ICIs are not effective in all patients, with only 10%–20% of patients with non-small-cell lung cancer (NSCLC) demonstrating long-term survival [4,5]. Therefore, prognostic markers in ICI therapy are required.

The expression of programmed cell death ligand 1 (PD-L1) in tumors was utilized as a predictive and prognostic marker of anti-PD-1/L1 antibodies [6]. Several clinical trials have revealed the effectiveness of anti-PD-1/L1 antibodies in patients with high PD-L1 expression; however, it demonstrated lower efficacy in patients with PD-L1 expression <50% [5,7,8,9,10]. However, PD-L1 expression alone is an insufficient prognostic marker. Even in patients with a PD-L1 of <50%, some respond to anti-PD-1/L1 antibody monotherapy and achieve long-term survival. Therefore, additional prognostic markers are necessary to deliver precision medicine for patients with NSCLC.

Several clinical trials of anti-PD-1/L1 antibody therapy have demonstrated relatively worse efficacy and prognosis in squamous NSCLC subtypes compared with non-squamous types [5,11]. In clinical practice, serum tumor markers are useful for estimating histological types. Serum cytokeratin fraction 21-1 (CYFRA 21-1) is a fragment of cytokeratin 19 and is a highly sensitive and specific NSCLC tumor marker for squamous subtypes [12,13]. Therefore, serum CYFRA 21-1, which represents a squamous cell subtype, may predict prognosis in anti-PD-1/L1 antibody therapy.

A few studies have indicated the association between serum CYFRA 21-1 and prognosis in patients receiving anti-PD-1/L1 antibody therapy [14,15]. However, their results were controversial. They analyzed NSCLC without stratification by histology or driver gene alteration, a negative indicator of anti-PD-1/L1 antibody therapy. Moreover, they analyzed NSCLC treated with anti-PD1/PD-L1 antibody monotherapy not including combination therapy with chemotherapy and/or anti-CTLA-4 antibodies, which are the standard treatments for NSCLC, particularly with PD-L1 expression <50% [16].

Therefore, this study aimed to investigate serum CYFRA 21-1 as a prognostic predictor in patients with NSCLC who received anti-PD-1/L1 antibodies. NSCLC with driver gene alterations were excluded, and the analysis was stratified by histology and treatment regimen. We found that serum CYFRA 21-1 is a poor prognostic marker for patients with NSCLC receiving anti-PD-1/PD-L1 antibody treatment. Serum CYFRA 21-1 may be useful for precision medicine with anti-PD-1/PD-L1 antibody treatment.

## 2. Materials and Methods

The primary endpoint was serum CYFRA 21-1 as a prognostic factor for anti-PD-1/L1 antibody therapy in patients with NSCLC without or with unknown driver gene alterations. We excluded patients with NSCLC harboring driver gene alterations, who typically respond well to molecular targeted therapy with a more limited response to anti-PD-1/PD-L1 antibody treatment [17,18]. Data from patients with NSCLC receiving anti-PD-1/L1 antibodies throughout their treatment history were retrospectively obtained.

All patients with a Union for International Cancer Control-Eighth Edition classification were stage IVA or IVB. Patients exhibiting postoperative recurrence were restaged before first-line therapy. Patients received at least one course of nivolumab, pembrolizumab, atezolizumab, durvalumab, or ipilimumab. Data on patient characteristics (age, sex, performance status [PS], smoking history, histology, PD-L1 expression, stage, ICI regimen, treatment line, serum CYFRA 21-1 level, and serum carcinoembryonic antigen [CEA] level) were also obtained.

This study was approved by the Institutional Review Board of our hospital (2023-GB-181, 2024-GB-011), which waived the need for informed consent because of the retrospective design and an opt-out method.

Clinical tests approved by the Pharmaceuticals and Medical Devices Agency, including the Lumipulse Presto CYFRA 21-1 chemiluminescent enzyme immunoassay (FUJIREBIO) and the Alinity I CEA chemiluminescence immunoassay (Abbott Japan), were performed to measure serum CYFRA 21-1 and CEA levels. Patients were categorized as having high or normal serum CYFRA 21-1 and CEA levels with cutoffs of 3.5 and 5.0 ng/mL, respectively, based on institutional criteria.

Fisher’s exact test was used for all analyses of categorical variables. Overall survival (OS) indicates the time from the initiation of first-line therapy to death. Progression-free survival (PFS) was defined as the time from the initiation of first-line therapy to disease progression or death. The Kaplan–Meier method was utilized to establish OS and PFS, and a log-rank test was conducted. We calculated variance inflation factors (VIFs) for the diagnosis of collinearity. The Cox proportional hazards model was used for univariate and multivariate analyses. Variables with *p*-values of <0.05 in the univariate analysis were included in the multivariate analysis. A *p*-value less than 0.05 was considered statistically significant. IBM SPSS For Windows version 29 (IBM Corp., Armonk, NY, USA) was used for all analyses.

## 3. Results

### 3.1. Patient Characteristics

**Figure 1:**
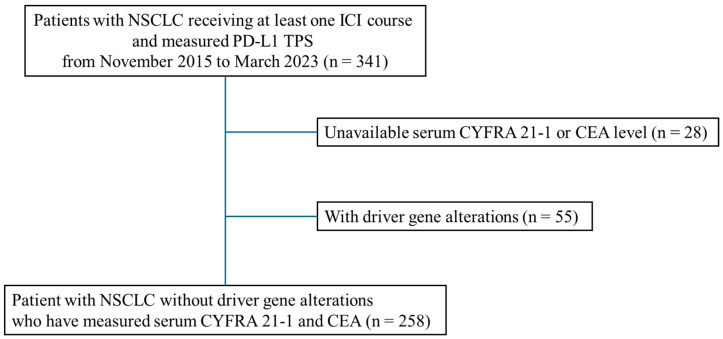
Patient flow.

From November 2015 to March 2023, 341 patients with NSCLC received at least one ICI course, and their PD-L1 tumor proportion score was measured. Moreover, 83 patients (28 with unavailable serum CYFRA 21-1 or CEA measurement, and 55 with driver gene alterations (EGFR, ALK, ROS1, RET, BRAF, and MET)) were excluded. Thus, 258 patients were included (Figure 1).

Of 258 patients, 47 and 211 had squamous and non-squamous NSCLC, respectively. Serum CYFRA 21-1 and CEA were positive in 117 (45.3%) and 131 (50.8%) patients, respectively. Table 1 presents patients’ characteristics based on histology, serum CYFRA 21-1, and CEA levels. The number of male patients, as well as the number of patients treated with anti-PD-1/L1 antibodies only, was significantly higher in the squamous NSCLC group than in the non-squamous NSCLC group. The high-serum-CYFRA 21-1 group exhibited significantly more patients with PS of ≥2, stage IVB, ICI as first-line therapy, and treated with combination therapy compared to the normal-serum-CYFRA 21-1 group. The high-serum-CEA group exhibited significantly more patients with a PD-L1 of <50% and treated with combination therapy compared to the normal-serum-CEA group (Table 1). The details of the combination therapies provided to the study cohort are as follows: 74 patients received anti-PD1/PD-L1 antibodies in combination with chemotherapy, 55 patients received combination immunotherapy with anti-PD-1/PD-L1 and anti-CTLA-4 antibodies, and the remaining 22 patients received anti-PD-1/PD-L1 antibodies in combination with clinical trial drugs.

### 3.2. OS Between Histology Types, High and Normal Serum CYFRA 21-1 Levels, and CEA Levels

**Figure 2:**
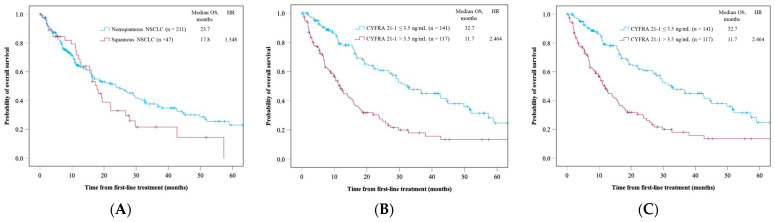
Overall survival of all patients in (**A**) histology, (**B**) serum CYFRA 21-1 level, and (**C**) serum CEA level. Time is expressed in months.

The squamous NSCLC group demonstrated a shorter OS than the non-squamous NSCLC group (median OS [mOS], 17.8 vs. 23.7 months, *p* = 0.141) (Figure 2A). The high-serum-CYFRA 21-1 group showed a significantly shorter OS than the normal-serum-CYFRA 21–1 group (mOS, 11.7 vs. 32.7 months, *p* < 0.005) (Figure 2B). The high-CEA group exhibited a significantly shorter OS than the normal-serum-CEA group (mOS, 15.8 vs. 29.7 months, *p* < 0.005) (Figure 2C).

### 3.3. Univariate and Multivariate Analyses of Variable Factors of OS

The univariate analysis of OS was conducted based on patient characteristics and indicated PS, smoking history, PD-L1 expression, stage, serum CYFRA 21-1 levels, and serum CEA levels as significant prognostic factors (hazard ratio [HR] with 95% confidence interval [CI]: 2.95 [1.94–4.50], 0.64 [0.42–0.99], 0.57 [0.40–0.81], 1.54 [1.11–2.14], 2.47 [1.77–3.43], and 1.72 [1.24–2.39], respectively). Multivariate analysis using factors that were significant in the univariate analysis identified a PS of ≥2, a PD-L1 expression of ≥50%, and high serum CYFRA 21-1 levels as independent prognostic factors (HR with 95% CI, 2.48 [1.58–3.88], 0.54 [0.38–0.78], and 1.99 [1.38–2.88], respectively) (Table 2). The HR of serum CYFRA 21-1 as a predictor of OS was comparable to that of the PD-L1 expression. In contrast, CEA was not a significant prognostic factor of OS (1.36 [0.97–1.91], *p* = 0.072). VIFs ranged from 1.012 to 1.177, indicating no significant collinearity between the covariates.

### 3.4. OS Between High and Normal Serum CYFRA 21-1 Levels in the Non-Squamous NSCLC Group

**Figure 3:**
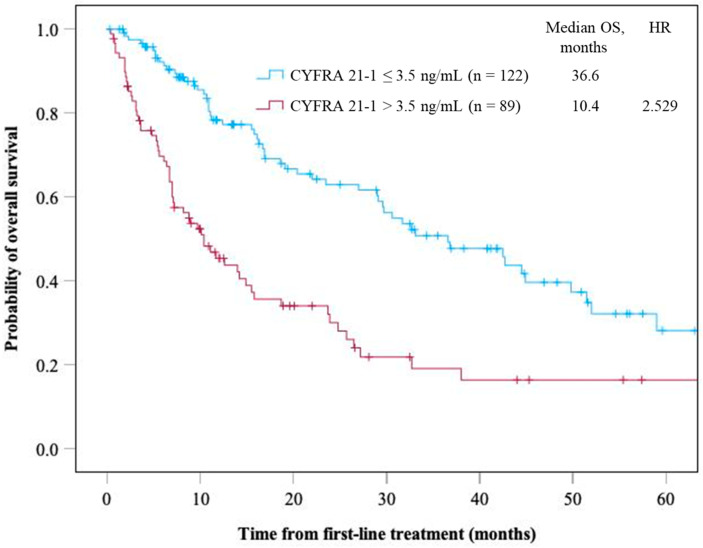
Overall survival of patients with non-squamous NSCLC based on the serum CYFRA 21-1 level. Time is expressed in months.

Serum CYFRA 21-1 was investigated as a prognostic marker in a subgroup with non-squamous NSCLC, and 42.2% (89/211 patients) had serum CYFRA 21-1 positivity. Supplementary Table S1 demonstrates the characteristics of patients with non-squamous NSCLC, indicating that significantly more patients with non-squamous NSCLC with high serum CYFRA 21-1 levels had a PS of ≥2, stage IVB, and ICI as a first-line therapy and were treated with ICI monotherapy compared to those with normal serum CYFRA 21-1 levels. The non-squamous NSCLC group with high serum CYFRA 21-1 levels demonstrated a significantly shorter OS than the non-squamous NSCLC group with normal serum CYFRA 21-1 levels (mOS, 10.4 vs. 36.6 months, *p* < 0.005) (Figure 3).

### 3.5. OS Between High and Normal Serum CYFRA 21-1 Levels in Different ICI Treatment Regimens

**Figure 4:**
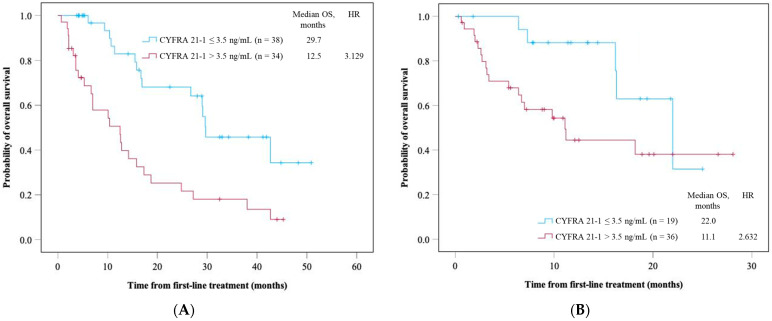
Overall survival of patients receiving the combination therapy of ICIs and (**A**) chemotherapy and (**B**) anti-CTLA-4 antibodies as first-line therapy in serum CYFRA 21-1 level. Time is expressed in months.

In this study, 72 patients were treated with the anti-PD-1/L1 antibody and chemotherapy combination as first-line therapy. Among them, 34 patients exhibited high serum CYFRA 21-1 levels. The high-serum-CYFRA 21-1 group demonstrated a significantly shorter OS than those in the normal-serum-CYFRA 21-1 group (mOS: 12.5 vs. 29.7 months, *p* < 0.005) (Figure 4A). The PFS was significantly shorter in the high-serum-CYFRA 21-1 group than in the normal-serum-CYFRA 21-1 group (median PFS: 4.9 vs. 7.7 months, *p* = 0.034) (Supplementary Figure S1A). Moreover, 55 patients were treated with anti-CTLA antibody combination therapy. Among them, 36 patients exhibited high serum CYFRA 21-1 levels. This high-serum-CYFRA 21-1 group demonstrated a significantly shorter OS than the normal-serum-CYFRA 21-1 group (mOS, 11.1 vs. 22.0 months, *p* = 0.048) (Figure 4B). The PFS was significantly shorter in the high-serum-CYFRA 21-1 group than in the normal-serum-CYFRA 21-1 group (median PFS: 3.5 vs. 8.1 months, *p* = 0.236) (Supplementary Figure S1B). 

We also evaluated the prognostic value of serum CYFRA 21-1 level in patients with NSCLC harboring driver gene alterations, which are shown in Supplementary Manuscript and Supplementary Figure S2.

## 4. Discussion

In this study, we investigated serum CYFRA 21-1 as a prognostic marker in anti-PD-1/PD-L1 antibody treatment. Patients with high serum CYFRA 21-1 levels demonstrated a significantly shorter OS than those with normal serum CYFRA 21-1 levels. In the multivariate analysis, high serum CYFRA 21-1 level was determined as an independent poor prognostic factor.

Generally, NSCLC prognosis and chemotherapy efficacy depend on the histological types [19,20]. Moreover, histology types are crucial prognostic factors in ICI therapy. Previous clinical trials have revealed that among those taking anti-PD-1/L1 antibody monotherapy and combination treatments with chemotherapy, the squamous NSCLC group demonstrated a shorter OS than the non-squamous NSCLC group [9,10,21,22,23,24]. Serum tumor markers are crucial in estimating histological types in clinical practice. CYFRA 21-1 is a highly sensitive and specific NSCLC tumor marker, particularly for squamous subtypes [13]. The sensitivity and specificity of serum CYFRA 21-1 for squamous NSCLC were 0.68 and 0.94, respectively [12]. In the present study, serum CYFRA 21-1 levels were elevated in 59.8% of the patients with squamous NSCLC, which was higher than that observed among the patients with non-squamous NSCLC (42.2%). Therefore, serum CYFRA 21-1 is considered a prognostic factor to substitute squamous cell histology type.

Moreover, serum tumor markers could be a better prognostic factor than histological type. This study indicated that high serum CYFRA 21-1 is an independent poor prognostic factor, different from the squamous cell histology. Tumor heterogeneity may have influenced the results. Differentiating the squamous or non-squamous NSCLC in patients with advanced NSCLC using small biopsy samples is sometimes challenging because of tumor heterogeneity [25]. Conversely, serum tumor markers reflect the whole tumor lesions and are unaffected by tumor heterogeneity [26].

Histology, smoking status, performance status, and other variables that are considered prognostic factors in anti-PD-1/PD-L1 treatment might be potential confounders in the analyses evaluating the prognostic significance of the serum CYFRA 21-1 level [27,28]. Therefore, we conducted multivariate analysis, which revealed CYFRA 21-1 level as an independent prognostic factor.

A few studies have indicated the association between serum CYFRA 21-1 levels and prognosis in patients receiving anti-PD-1/L1 antibodies. Shirasu et al. reported a long progression-free survival in patients with lung adenocarcinoma treated with nivolumab as a second-line therapy or a later-line therapy in patients with high CYFRA 21-1 (≥2.2 ng/mL) [14]. However, they did not examine the expression of PD-L1, the standard prognostic marker in anti-PD-1/L1 antibody treatment, and included driver gene alteration-positive NSCLC, which is less effective than anti-PD-1/L1 antibodies [17], which may cause a discrepancy in the results. Moreover, the majority of the patients (83.7%) received anti-PD-1/PD-L1 antibodies as a first-line treatment in the present study. Dall’Olio et al. revealed short OS in patients with high CYFRA 21-1 levels (>8 ng/mL) treated with anti-PD-1/L1 antibodies [15]. However, this report analyzed only anti-PD-1/L1 antibody monotherapy. Therefore, in the present study, serum CYFRA 21-1 was analyzed as a prognostic marker in various ICI treatment regimens, and the prognosis difference with serum CYFRA 21-1 levels in each treatment was determined.

Moreover, serum CYFRA 21-1 was examined as a prognostic marker of non-squamous NSCLC, which is different from the past report [15]. The present study revealed that high serum CYFRA 21-1 levels were significantly associated with poor prognosis, even in patients with non-squamous NSCLC. Considering the tumor heterogeneity in squamous and non-squamous NSCLC, classifying patients with NSCLC by histology and further categorizing them by serum tumor markers may be useful in precision medicine.

A high tumor burden was one of the poor prognostic factors in ICI therapy [29], and tumor markers correlated with the tumor volume [30]. A high serum CYFRA 21-1 level only indicates a high tumor burden, which may have correlated with poor prognosis. Therefore, a multivariate analysis, including stage IVA or IVB, which represents tumor burden, was performed and revealed high serum CYFRA 21-1 level as an independent poor prognostic factor.

This study has some other limitations. First, the possibility of biases cannot be ruled out, because of the retrospective design of this single-center study, which limits the generalizability of our findings. Single-center analysis might introduce patient selection bias with unmeasured reasons. Although we conducted multivariate analysis with multiple prognostic factors to minimize such biases, a selection bias as well as other potential biases inherent to retrospective studies persist. Furthermore, although we aimed to adjust for known confounders, it is possible that other unmeasured variables might have impacted our results. Thus, the current study findings should be interpreted with caution based on this limitation. Future multi-center studies with larger sample sizes are warranted to provide more robust evidence and to support the generalizability of our findings by reducing study-site-specific biases. Second, the retrospective study design precluded external validation analyses. The cutoffs used for tumor markers were based on institutional criteria, and prospective studies are warranted to elucidate precise cutoffs to predict the effect of anti-PD-1/PD-L1 antibodies and to confirm the utility of serum CYFRA 21-1 level across different clinical settings. Third, this study only investigated prognostic factors of patients receiving ICIs, so the assessment of ICI efficacy prediction is insufficient. Therefore, analysis of the overall response and progression-free survival in a single ICI regimen with a large number of patients is warranted. Fourth, we also demonstrated the association between serum CYFRA 21-1 levels and prognosis in patients treated with anti-PD-1/PD-L1 antibodies. However, we did not evaluate the direct mechanisms by which serum CYFRA 21-1 levels were associated with worse prognosis in patients with NSCLC treated with anti-PD-1/PD-L1 antibodies, a limitation of our study. Further preclinical studies are warranted to evaluate this association. Fifth, some patients without available data on serum CYFRA 21-1 levels were excluded from this study, which might have introduced patient selection bias.

## 5. Conclusions

In this study, a high serum CYFRA 21-1 level was found to be a poor prognostic marker in patients with NSCLC receiving anti-PD-1/L1 antibodies even stratifying by histology or treatment regimen. Thus, serum CYFRA 21-1 may be useful for precision medicine with anti-PD-1/PD-L1 antibody treatment. The confirmation of our findings in larger studies will facilitate the utility of serum CYFRA 21-1 level as an important factor for patient stratification in clinical trials on immunotherapy.

## Figures and Tables

**Table 1:** Patient characteristics.

	Total	Histology			CYFRA 21-1			CEA		
		Non-Squamous NSCLC	Squamous NSCLC	*p*	≤3.5 ng/mL	>3.5 ng/mL	*p*	≤5.0 ng/mL	>5.0 ng/mL	*p*
Age, *n*										
<75	202 (78.3)	170 (80.6)	32 (68.1)	0.077	110 (78.0)	92 (78.6)	1.000	99 (78.0)	103 (78.6)	1.000
≥75	56 (21.7)	41 (19.4)	15 (31.9)		31 (22.0)	25 (21.4)		28 (22.0)	28 (21.4)	
Sex, *n*.										
Male	201 (77.9)	158 (74.9)	43 (91.5)	0.011	111 (78.7)	90 (76.9)	0.764	103 (81.1)	98 (74.8)	0.234
Female	57 (22.1)	53 (25.1)	4 (8.5)		30 (21.3)	27 (23.1)		24 (18.9)	33 (25.1)	
PS, *n*										
0–1	224 (86.8)	184 (87.2)	40 (85.1)	0.641	133 (94.3)	91 (77.8)	<0.001	114 (89.8)	110 (84.0)	0.199
≥2	34 (13.2)	27 (12.8)	7 (14.9)		8 (5.7)	26 (22.2)		13 (10.2)	21 (16.0)	
Smoking status, *n*										
Never smoked	36 (14.0)	33 (15.6)	3 (6.4)	0.108	16 (11.3)	20 (17.1)	0.209	17 (13.4)	19 (14.5)	0.858
Current or former	222 (86.0)	178 (84.4)	44 (93.6)		125 (88.7)	97 (82.9)		110 (86.6)	112 (85.5)	
PD-L1, *n*										
<50%	157 (60.9)	128 (60.7)	29 (61.7)	1.00	80 (56.7)	77 (65.8)	0.159	65 (51.2)	92 (70.2)	0.002
≥50%	101 (39.1)	83 (39.3)	18 (38.3)		61 (43.3)	40 (34.2)		62 (48.8)	39 (29.8)	
Stage, *n*										
IVA	116 (45.0)	90 (42.7)	26 (55.3)	0.144	82 (58.2)	34 (29.1)	<0.001	64 (50.4)	52 (39.7)	0.104
IVB	142 (55.0)	121 (57.3)	21 (44.7)		59 (41.8)	83 (70.9)		63 (49.6)	79 (60.3)	
Treatment line with ICIs, *n*										
First-line therapy	216 (83.7)	178 (84.4)	38 (80.9)	0.520	109 (77.3)	107 (91.5)	<0.005	102 (80.3)	114 (87.0)	0.178
Second-line or later	42 (16.3)	33 (15.6)	9 (19.1)		32 (22.7)	10 (8.5)		25 (19.7)	17 (13.0)	
Therapy, *n*.										
Anti-PD-1/L1 Ab only	107 (41.5)	81 (38.4)	26 (55.3)	0.049	68 (48.2)	39 (33.3)	0.016	61 (48.0)	46 (35.1)	0.043
Combination therapy	151 (58.5)	130 (61.6)	21 (44.7)		73 (51.8)	78 (66.7)		66 (52.0)	85 (64.9)	

Ab, antibody; CEA, carcinoembryonic antigen; CYFRA 21-1, cytokeratin fraction 21–1; ICI, immune checkpoint inhibitor; NSCLC, non-small-cell lung cancer; PD-L1, programmed cell death ligand 1; PS, performance status.

**Table 2:** Clinical factors associated with overall survival.

		Univariate Analysis		Multivariate Analysis	
		HR (95% CI)	*p*	HR (95% CI)	*p*
Age, year	<75	Reference			
	≥75	1.37 (0.91–2.06)	0.137		
Sex	Female	Reference			
	Male	0.93 (0.64–1.37)	0.727		
PS	0–1	Reference		Reference	
	≥2	2.95 (1.94–4.50)	<0.005	2.48 (1.58–3.88)	<0.005
Smoking status	Never smoker	Reference		Reference	
	Smoker	0.64 (0.42–0.99)	0.046	0.74 (0.48–1.15)	0.181
Histology	Non-squamous	Reference			
	Squamous	1.35 (0.90–2.01)	0.143		
PD-L1 status	<50%	Reference		Reference	
	≥50%	0.57 (0.41–0.81)	0.002	0.54 (0.38–0.78)	<0.005
Stage	IVA	Reference		Reference	
	IVB	1.54 (1.11–2.14)	0.010	1.16 (0.82–1.66)	0.403
CYFRA 21-1	≤3.5 ng/mL	Reference		Reference	
	>3.5 ng/mL	2.47 (1.77–3.43)	<0.005	1.99 (1.38–2.88)	<0.005
CEA	≤5.0 ng/mL	Reference		Reference	
	>5.0 ng/mL	1.72 (1.24–2.39)	<0.005	1.36 (0.97–1.91)	0.072

CEA, carcinoembryonic antigen; CI, confidence interval; CYFRA 21-1, cytokeratin fraction 21–1; HR, hazard ratio; PD-L1, programmed cell death ligand 1; PS, performance status.

## Data Availability

The datasets generated and/or analyzed during the current study are available from the corresponding author upon reasonable request.

## Supplementary Materials

The following supporting information can be downloaded at https://www.mdpi.com/article/10.3390/cancers16213712/s1. Table S1: The characteristics of patients with non-squamous NSCLC. Figure S1. PFS of patients receiving the combination therapy of ICIs and (A) chemo-therapy and (B) anti-CTLA-4 antibody as first-line therapy in serum CYFRA 21-1 level. Time is expressed in months; Figure S2. Overall survival of patients with driver gene alteration based on the serum CYFRA 21-1 level. Time is expressed in months.
