# Supplementary material for: Serum CYFRA 21-1 as a Prognostic Marker in Non-Small-Cell Lung Cancer Patients Treated with Immune Checkpoint Inhibitors
Source: Cancers (Basel). 2024 Nov 4;16(21):3712. doi: 10.3390/cancers16213712 (PMC11544981; doi:10.3390/cancers16213712)
Supplement: Supplementary file 1 [file cancers-16-03712-s001.zip › cancers-3260897-supplementary.pdf]

Supplementary Table S1. The characteristics of patients with nonsquamous NSCLC.

	CYFRA 21-1		
	≤3.5 ng/mL	>3.5 ng/mL	*p*
Age, n. (%)			
<75	98 (80.3)	72 (80.9)	1.000
≥75	24 (19.7)	17 (19.1)	
Sex, n. (%)			
Male	95 (77.9)	63 (70.8)	0.263
Female	27 (22.1)	26 (29.2)	
PS, n. (%)			
0–1	115 (94.3)	69 (77.5)	<0.005
≥2	7 (5.7)	20 (22.5)	
Smoking status, n (%)			
Never smoked	14 (11.5)	69 (77.5)	0.057
Current or former smokers	108 (88.5)	20 (22.5)	
PD-L1 status, n (%)			
<50%	70 (57.4)	58 (65.2)	0.318
≥50%	52 (42.6)	31 (34.8)	
Stage, n (%)			
ⅣA	68 (55.7)	22 (24.7)	<0.005
ⅣB	54 (44.3)	67 (75.3)	
Treatment line with ICIs, n (%)			
First-line therapy	94 (77.0)	84 (94.4)	<0.005
Second-line therapy or later	28 (23.0)	5 (5.6)	
Therapy, n. (%)			
Only anti-PD-1/L1 ab	66 (54.1)	64 (71.9)	0.010
Chemotherapy combined	56 (45.9)	25 (28.1)	

Ab: antibody; PS: performance status; ICI: immune checkpoint inhibitor; PS: performance status; PD-L1: programmed cell death ligand 1.

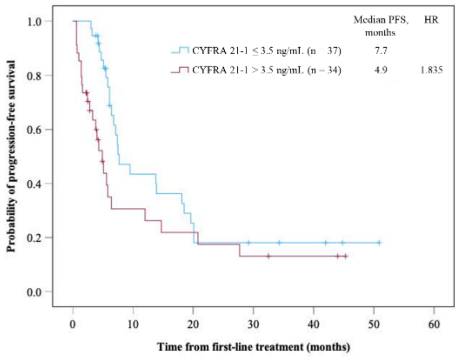

Supplementary Figure S1A

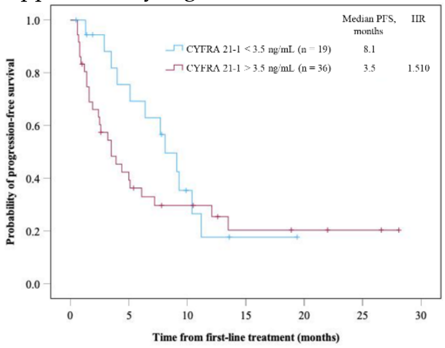

Supplementary Figure S1B

Supplementary Figure S1. PFS of patients receiving the combination therapy of ICIs and (**A**) chemo-therapy and (**B**) anti-CTLA-4 antibody as first-line therapy in serum CYFRA 21-1 level. Time is expressed in months.

Supplementary Manuscript

We also evaluated the prognostic value of serum CYFRA 21-1 level in patients with NSCLC harboring driver gene alterations and found that 34.5% (19/55) of the patients had serum CYFRA 21-1 positivity. Additionally, the OS was significantly shorter in patients with high serum CYFRA 21-1 levels than in those with normal serum CYFRA 21-1 levels within this group (mOS: 19.8 vs. 52.6 months, p = 0.091) (Supplementary Figure S2).

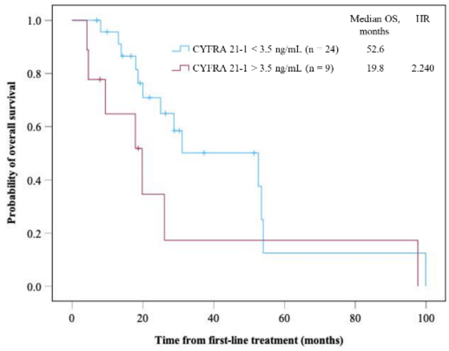

Supplementary Figure S2. Overall survival of patients with driver gene alteration based on the serum CYFRA 21-1 level. Time is expressed in months.
